# Risk Factors for Postnatal Growth Faltering and Undernutrition at Discharge in Very Preterm Infants: A Retrospective Study Applying the ESPGHAN Consensus Definitions

**DOI:** 10.3390/nu18020286

**Published:** 2026-01-16

**Authors:** Isadora Beghetti, Dalila Magno, Ettore Benvenuti, Arianna Aceti, Luigi Tommaso Corvaglia

**Affiliations:** 1 Department of Medical and Surgical Sciences, University of Bologna, 40138 Bologna, Italy; isadora.beghetti@unibo.it (I.B.); dalila.magno@studio.unibo.it (D.M.); luigi.corvaglia@unibo.it (L.T.C.); 2 Neonatal Intensive Care Unit, S. Orsola Hospital, IRCCS Azienda Ospedaliero Universitaria di Bologna, 40138 Bologna, Italy

**Keywords:** preterm infants, growth faltering, undernutrition, ESPGHAN, Intergrowth 21st, neonatal nutrition, growth restriction, VLBW

## Abstract

Background: Postnatal growth failure in very preterm infants remains a major concern in neonatal care and clinical management is complicated by the lack of a standardized definition. This study aims to identify risk factors for growth faltering (GF) and undernutrition (UN) at hospital discharge, defined according to the latest consensus definitions established by the European Society for Paediatric Gastroenterology, Hepatology and Nutrition (ESPGHAN). Methods: We conducted a retrospective observational study of 416 preterm infants (gestational age < 32 weeks and/or birth weight < 1500 g). Growth was monitored using the Intergrowth 21st standards. In line with ESPGHAN criteria, GF was defined longitudinally as a weight for age (WFA) z-score decline ≥ 1 SD from birth, while UN was defined cross-sectionally as a WFA or length for age z-score < −2 SD at discharge. Logistic regression models were used to determine independent predictors for both growth phenotypes. Results: At discharge, the prevalence of GF and UN was 45.3% and 33.1%, respectively. In infants born without growth restriction (GR), UN was almost entirely driven by GF (89.7%). In contrast, 85.5% of infants born with GR remained undernourished at discharge. Multivariate analysis identified bronchopulmonary dysplasia and higher maximal postnatal weight loss as major independent risk factors for GF, while female sex and human milk feeding at discharge were associated with a lower risk of GF. For infants born with adequate weight, maternal hypertension, extremely low birth weight, and the co-occurrence of GF were the strongest predictors of UN. Conclusions: Nearly half of very preterm infants experience significant growth impairment before discharge. By assessing the dynamic process of GF and the static endpoint of UN, we identified distinct clinical trajectories. Standardized ESPGHAN criteria allow for the identification of high-risk “phenotypes”—particularly those with GR at birth or severe neonatal morbidity—enabling more targeted and intensive nutritional management during the critical developmental window.

## 1. Introduction

Postnatal growth failure in preterm infants remains a major concern in neonatal care, reflecting suboptimal postnatal growth that can adversely affect long-term health and neurodevelopment. The most recent consensus from the European Society for Paediatric Gastroenterology, Hepatology and Nutrition (ESPGHAN) highlights the necessity of continuous growth monitoring and individualized nutritional management after hospital discharge. Attention is required for infants who experience significant drops in weight or length z-scores exceeding 2 standard deviations (SDs), as these infants require tailored nutritional support to optimize outcomes [1].

Postnatal growth failure is variably defined in the literature, with two principal approaches: cross-sectional definitions (weight < 10th centile or <−1/−2 SDs at a specific postnatal time point, such as discharge or term-equivalent age) and longitudinal definitions (a decrease in weight z-score > 1 or >2 SD from birth to a defined time point) [2,3,4]. Different growth charts (e.g., Fenton, Intergrowth 21st) and timing of assessment further influence prevalence estimates and risk stratification, leading to significant heterogeneity in reported rates, and complicating both clinical management and research comparisons [5,6,7].

The importance of postnatal growth failure lies in its association with adverse outcomes, including impaired neurodevelopment, persistent growth deficits, and increased cardiometabolic risk in childhood [4,8,9,10]. Longitudinal definitions, which account for changes in growth trajectory, appear to better predict long-term outcomes than cross-sectional definitions [2,4]. The lack of a universally accepted definition underscores the need for standardized criteria to facilitate early identification and intervention for at-risk infants.

While risk factors associated with the traditional cross-sectional definition are extensively studied, including lower gestational age (GA) and birth weight (BW), male sex, adverse perinatal factors, severe neonatal morbidities, and nutritional deficits [11,12,13,14], determinants for the longitudinal definition remain less clearly characterized, despite the emerging consensus that longitudinal assessment provides a superior reflection of dynamic growth trajectories.

Therefore, the present study aims to describe the risk factors for growth faltering (GF) and undernutrition (UN) at discharge based on the refined definitions of postnatal growth failure established by the recent ESPGHAN position paper, with the ultimate goal of facilitating early identification of infants who require a targeted and intensive nutritional management.

## 2. Materials and Methods

A retrospective, observational study was conducted at a level IV Neonatal Intensive Care Unit (NICU), located at IRCCS AOU Bologna, Italy, looking at predictors of impaired growth at discharge in preterm infants.

The study was conducted in accordance with principles and standards of the Helsinki Declaration. Clinical and growth data of infants participating in a prospective study on preterm infants’ neurodevelopment were collected retrospectively (study code: 76/2013/U/Sper/AOUBo, approved by the local Institutional Review Board—CE AVEC, Bologna, Italy). Given the retrospective nature of the present study, additional informed consent from individual participants was not required.

Infants born below <32 weeks GA and/or 1500 g BW between 2013 and 2019 were recruited, and the following clinical data were collected:

- Prenatal data: maternal variables which could be related to postnatal infant growth were included in the database: chorioamnionitis, maternal hypertension, prenatal steroid and magnesium sulphate prophylaxis. As for fetal variables, twin status and intrauterine growth restriction (IUGR) diagnosis [15] were included, as deemed relevant for further growth evaluation.
- Perinatal data: the following perinatal variables were collected: type of delivery, infant sex, GA, 5’ Apgar score, and anthropometry at birth (BW, length and head circumference [HC], with their respective centile and z-scores).
- Neonatal data: the presence of the main neonatal comorbidities was assessed by checking the infants’ medical records. Specifically, the following variables were recorded and staged according to the definitions provided by the Vermont Oxford Network [16]: intraventricular hemorrhage (IVH), periventricular leukomalacia (PVL), early- and late-onset sepsis (EOS and LOS), necrotizing enterocolitis (NEC), and patent ductus arteriosus (PDA). Bronchopulmonary dysplasia (BPD) was diagnosed and staged according to the definition proposed in 2019 by Jensen et al. [17].
- Discharge data: length of hospital stay and postmenstrual age (PMA) at discharge were recorded. Provision of human milk (HM) at discharge was recorded. Growth was assessed using the Intergrowth 21st charts for postnatal growth of preterm infants [18], in line with international consensus and following the guidelines for monitoring growth in preterm infants issued by the Italian Society of Neonatology [19], which recommend using the Intergrowth 21st charts up to six months corrected age, and the WHO growth charts from then on.

At each timepoint, the quality of growth was classified according to the definitions proposed by the recent paper by the ESPGHAN [1]: specifically,

- At birth, infants were classified as small for gestational age (SGA) when the BW centile was <3rd, while a definition of growth restriction (GR) was made if BW was <3rd or <−2 SD or in the presence of at least three of the following: BW < 10th percentile, HC at birth < 10th centile, length at birth < 10th centile, prenatal diagnosis of IUGR or maternal hypertension/preeclampsia [20].
- At discharge, GF was defined as a fall in weight for age (WFA) z-score ≥ 1.0 from birth, while undernutrition (UN) as a weight for age (WFA) or length for age (LFA) z-score < −2 SDs.

### 2.1. Nutritional and Clinical Management

Management followed a standardized institutional protocol, which did not change over the study period. Parenteral nutrition was initiated immediately after birth, with a starting fluid intake of 80 mL/kg/day. Enteral feeding was typically introduced within the first 24–48 h of life as minimal enteral feeding (MEF). Own mother’s milk was the first choice for enteral feeding, supplemented by donor human milk in case of insufficient maternal supply or unavailability. In accordance with the clinical protocol in use during the study period, the duration of MEF and the subsequent rate of feed advancement were determined by individual risk factors and feeding tolerance. Once advancing, feeds were typically increased by 15–25 mL/kg/day. Human milk fortification (using bovine-based multi-nutrient fortifier) was initiated once an enteral intake of 100 mL/kg/day was reached and tolerated.

Regarding environmental control, infants were managed in incubators with servo-controlled humidity to minimize transepidermal water loss. Humidity was set between 70% and 90%, with higher levels targeted for infants with lower gestational ages. These levels were typically maintained for the first week, then gradually tapered based on skin maturity and fluid balance.

### 2.2. Statistical Analysis

All statistical analyses were carried out using IBM SPSS Statistics for Windows, Version 28.0 (IBM Corp., Armonk, NY, USA). Data distribution was checked using the Kolmogorov–Smirnov test. As data did not follow a normal distribution, non-parametric tests were used. Continuous variables are thus presented as median (interquartile range [IQR]) and dichotomous variables as number (percentage).

Prenatal, perinatal and neonatal variables potentially related with GF and UN at discharge were first investigated within two separate univariate analyses in which the Mann–Whitney U test was used for continuous variables, and the chi-square test for dichotomous variables.

Before performing the multivariate analyses, potential collinearity between independent variables to be included in the regression models was checked using the Pearson correlation coefficient or the point-biserial correlation coefficient as appropriate. Correlations were defined as “strong” when correlation coefficients were above 0.6 (or below −0.6).

After assessment of collinearity, variables which proved to be significantly associated with GF or UN at discharge at the univariate analysis were included into separate logistic regression models aimed at identifying risk and protective factors for GF and UN.

Exploratory ROC analyses using the predicted probabilities derived from the multivariable regression models were performed to assess the ability of identified risk factors to discriminate infants at risk for GF and UN. Sensitivity and specificity of each model were calculated by assessing the largest Youden index.

A *p*-value < 0.05 was considered statistically significant.

## 3. Results

Four-hundred-forty-eight very low GA (VLGA) and/or VLBW infants, admitted at birth at the study NICU, were included in the study. Thirty-two infants died before discharge, and the remaining 416 infants constituted the study population.

Data about GF (defined as a fall in WFA z-score ≥ 1.0 from birth) and UN at discharge (defined as a WFA or LFA z-score < −2 SDs) were available for 402 infants (14 missing data). GF occurred in 182 (45.3%) infants and UN was present in 133 (33.1%) infants.

### 3.1. Growth Faltering at Discharge

Variables potentially related to GF at discharge are depicted in Table 1.

Specifically, at birth infants with GF were younger and smaller compared to those with adequate growth at discharge. Given the longitudinal nature of the measure, IUGR, GR at birth and SGA were less frequent in infants with GF. GF was more common in males and in those exhibiting a higher weight loss after birth; comorbidities associated with GF were IVH, PVL, LOS, BPD, PDA requiring treatment, and NEC stage ≥ 2. Infants with GF were discharged later and at a higher postmenstrual age and were less likely to receive HM at discharge compared to those with adequate growth.

Collinearity assessment revealed that growth parameters at birth (birth weight, length, and HC, and ELBW), GA, length of hospital stay and PMA at discharge were all strongly related, as well as IUGR, GR at birth and SGA. For this reason, ELBW was selected within the first group of variables, and GR at birth within the second, leading to a final regression model including GR at birth, ELBW, sex, IVH, LOS, BPD, PDA, NEC, weight loss after birth and HM at discharge (Table 2). A higher weight loss after birth and BPD were found to be significantly associated with an increased risk of GF at discharge; conversely, females and infants receiving HM at discharge were less likely to experience GF.

ROC analysis yielded an AUC of 0.762 (95% CI: 0.713–0.818), indicating acceptable discriminative ability. At the optimal cut-off (Youden index), sensitivity was 75% and specificity was 68%.

### 3.2. Undernutrition at Discharge

UN at discharge can result from different growth trajectories according to the nutritional status at birth: for this reason, two separate analyses were conducted to assess risk factors for UN in infants with GR at birth and in those with an adequate nutritional status at birth.

**Figure 1:**
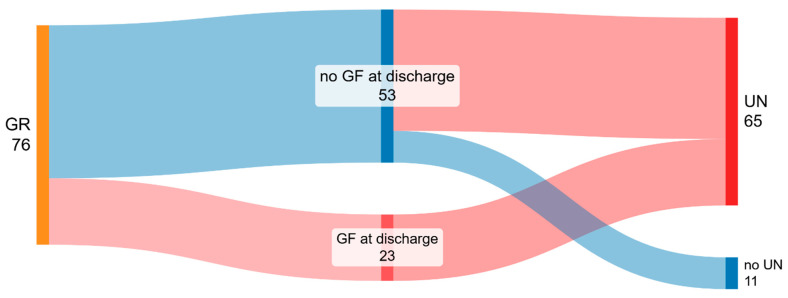
Growth trajectories of infants with growth restriction (GR) at birth. Sankey diagram represents subjects’ growth paths from birth to hospital discharge. Sankey diagram is a visualization technique that allows to represent flows between different entities and their magnitudes. Several entities (nodes) are represented by rectangles. The initial node on the left represents nutritional status at birth. The final nodes on the right depict growth assessment at discharge categorized as “growth faltering (GF)” and “no growth faltering” and further classified in “undernutrition (UN)” and “no undernutrition”. The size of each node reflects the number of infants within each growth phenotype group. The connecting arcs visualize the pathways of the infants. The width of each arc is proportional to the number of infants transitioning between groups, and the color of each arc corresponds to the target node. Labels’ dimensions are proportional to number of subjects in each node. Figure was made at SankeyMATIC.com (date of access 17 December 2025).

Seventy-six infants were diagnosed with GR at birth; among these, 65 (85.5%) had UN (WFA and/or LFA < −2 SDs) and 11 (14.5%) an adequate nutritional status at discharge. No difference in terms of GA or anthropometry at birth was documented between infants with and without UN at discharge. Infants with UN were more frequently males (57% in UN vs. 20% in the adequate nutrition group, *p* = 0.042). Among infants with UN, 23 (35.3%) also had GF at discharge (meaning that all the infants with both GR at birth and GF at discharge also had UN at discharge—Figure 1).

Infants with UN had a longer median hospital stay compared to those without UN (median 30 days [IQR 42] vs. 22.5 days [IQR 18]), though this difference was not statistically significant (*p* = 0.199). However, they were discharged at a significantly higher PMA (median 38.1 weeks [IQR 2.9] vs. 36.3 weeks [IQR 0.9], *p* = 0.006).

No regression analysis was performed in this subgroup of patients, given that only two variables (sex and GF) were significantly different between groups and the sample was relatively small.

**Figure 2:**
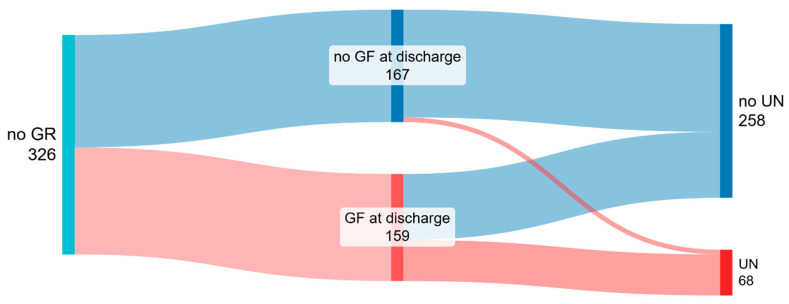
Growth trajectories of infants with adequate nutritional status (no growth restriction [GR]) at birth. In the depicted diagram, the initial node on the left represents nutritional status at birth. The final nodes on the right depict growth assessment at discharge categorized as “growth faltering (GF)” and “no growth faltering” and further classified in “undernutrition (UN)” and “no undernutrition”. Figure was made at SankeyMATIC.com (date of access 17 December 2025).

Three-hundred-twenty-six infants did not have GR at birth; among these, 68 (20.9%) had UN at discharge, and 258 (79.1%) an adequate nutritional status at discharge. Maternal hypertension was more frequent in infants with UN (32.8% vs. 20.6%, *p* = 0.046). No significant differences in GA were documented; infants with UN were smaller in terms of birth weight (median 890 g [IQR 438] vs. 1335.5 g [IQR 438], *p* < 0.001), length (median 34 cm [IQR 6] vs. 39 cm [IQR 4], *p* < 0.001), and HC (median 26 cm [IQR 5] vs. 28 cm [IQR 3], *p* < 0.001), and ELBW was more frequent (50% vs. 23%, *p* < 0.001). Infants with UN were more frequently males (66.2% in UN vs. 33.8% in the adequate nutrition group, *p* = 0.009). As for neonatal morbidity, infants with UN suffered more frequently of LOS (29.9% vs. 13.5%, *p* = 0.003), BPD (35.3% vs. 14.7%, *p* < 0.001). HM feeding at discharge was less frequent in infants with UN (67.6% vs. 80.2%, *p* = 0.033). Among infants with UN, 61 (89.7%) also had GF at discharge (Figure 2).

Infants with UN were discharged later (median 78.7 days [IQR 78] vs. 42 days [IQR 25], *p* < 0.001) and at a higher PMA (median 37.8 weeks [IQR 6.3] vs. 35.7 weeks [IQR 2.1], *p* < 0.001).

After assessment of collinearity, variables which proved to better predict UN at discharge were maternal hypertension, ELBW, and the co-occurrence of GF at discharge (Table 3).

**Figure 3:**
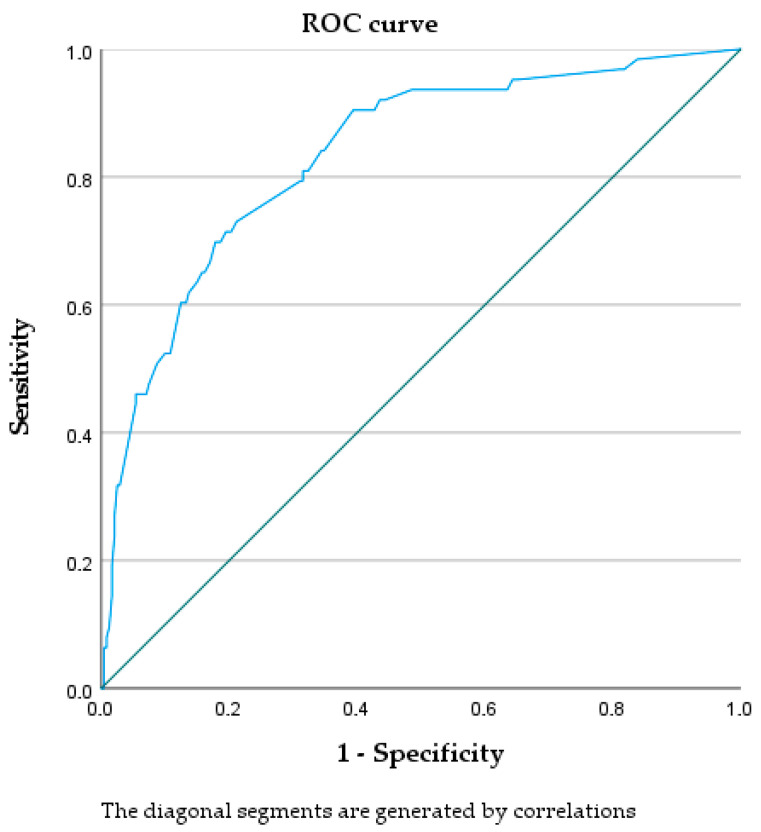
ROC curve assessing the ability of risk factors identified through regression analysis to discriminate, among infants without growth restriction at birth, those at risk for undernutrition.

ROC analysis (Figure 3) yielded an AUC of 0.834 (95% CI: 0.777–0.890), indicating good discriminative ability. At the optimal cut-off (Youden index), sensitivity was 70% and specificity was 82%.

## 4. Discussion

The primary objective of this study was to characterize postnatal growth impairment in VLGA/VLBW infants using the refined definitions proposed by the recent ESPGHAN position paper [1]. A major strength of our work is the adoption of these standardized criteria, which allow for a clear distinction between GF—a dynamic, longitudinal loss of growth momentum—and UN—a static measurement of poor nutritional status at a specific time point. Our results confirm that growth impairment at discharge remains highly prevalent in infants born below 32 weeks or 1500 g, occurring in almost half of our population, and highlight the distinct clinical trajectories that lead to these outcomes.

To accurately identify these infants, the choice of growth curves is paramount. We utilized the Intergrowth 21st charts, which, unlike older cross-sectional references, provide a prescriptive standard for how preterm infants should grow under optimal conditions [18,21]. Using shared prescriptive growth standards is essential for international benchmarking and for ensuring that growth targets in the NICU reflect longitudinal growth potential rather than cross-sectional references derived from anthropometrical parameters at birth [22].

Our data underscore that GF and UN definitions are not interchangeable. Actually, while GF is a strictly longitudinal definition, UN at discharge can be the result of at least three different growth trajectories [1]:

- Infants with an adequate nutritional status at birth, suffering from a significant GF during hospital stay leading to a discharge WFA and/or LFA < −2 SDs.
- Infants with GR who, despite an adequate postnatal growth, fail to reach a discharge WFA and/or LFA > −2 SDs.
- Infants with both GR and GF, leading to a discharge WFA and/or LFA < −2 SDs.

In addition, while UN at discharge is the clinical “endpoint”, GF represents a “process” leading to suboptimal growth. By distinguishing the two, we identified that for infants born with an adequate weight, UN is almost always the consequence of a significant longitudinal drop in WFA z-score. Conversely, for infants born with GR, UN is often a persistent state from birth. This distinction is vital for tailoring nutritional interventions, preventing the “slide” in the former and promoting safe “catch-up” in the latter.

Many risk factors identified for GF, such as BPD, are well-established in the literature due to increased metabolic demands and fluid restrictions [13,23,24,25]. In line with existing literature, we found that traditional markers of neonatal clinical severity—such as BPD and male sex—were significant predictors for GF [11,12,13]. In addition, in our study, maximal weight loss after birth emerged as a key independent risk factor for GF at discharge. This suggests that the nutritional debt incurred in the first days of life is difficult to recoup, setting the stage for a trajectory of sustained growth failure, and managing this early deficit remains a cornerstone of neonatal care [26,27,28].

Our analysis identified specific “red flag” categories that require maximum clinical surveillance: specifically, infants born with GR who subsequently suffer of GF are at the absolute highest risk for UN. In our cohort, all male infants who had both GR and GF remained undernourished at discharge. Infants born with adequate weight, but who are ELBW or born to mothers with hypertension, are also highly vulnerable. In these cases, maternal hypertension might reflect a compromised placental environment that, while not resulting in birth GR, impairs the metabolic status and physiological balance of the preterm infant [29,30].

We observed that infants with UN were less likely to be receiving HM at discharge, and this likely reflects a complex association between length of stay and HM provision to preterm infants [31,32,33]. Infants with UN often had more comorbidities and longer hospitalizations; it is well-documented that mothers of infants with prolonged NICU stays face significant challenges in maintaining milk expression. Thus, the lower rate of HM in the UN group may be a marker of early cessation of breastfeeding or milk expression. However, after controlling for confounders in our multivariate analysis, HM feeding did not have a negative impact on growth. This finding suggests that, while fortification remains essential to meet caloric and nutritional targets, the bioactive properties of HM may mitigate the downward shift in longitudinal growth trajectory [34].

Finally, it is important to recognize that anthropometric measurements provide only a surrogate assessment of nutritional status. While weight gain is the most commonly used metric, linear growth represents a more critical marker of nutritional adequacy and long-term health outcomes. As highlighted by the ESPGHAN Nutrition Committee, continuous monitoring of length is essential because linear growth deficits are strongly associated with impaired organ development and adverse neurocognitive outcomes [1,35,36,37,38]. Furthermore, preterm infants are at increased risk of developing an altered body composition at discharge, characterized by increased adiposity and reduced lean mass compared to term infants, even when weight gain appears adequate [39,40,41,42]. Although direct measures of body composition were unavailable in this retrospective cohort, the ESPGHAN definition of UN applied in our study explicitly incorporates linear growth failure. Since linear growth serves as a clinical proxy for protein accretion, by adhering to these criteria, we aimed to capture deficits in lean mass, ensuring that the identified high-risk phenotypes reflect also structural growth quality rather than weight status alone. Future prospective studies are needed to further characterize the detailed relationship between these standardized growth definitions and specific body composition outcomes.

Our observations support the potential value of early risk stratification to tailor nutritional management. Our data suggest that infants presenting with identifiable risk factors, specifically male sex, evolving BPD, excessive weight loss, ELBW, are particularly vulnerable to GF. Consequently, these high risk phenotypes may benefit from intensified nutritional surveillance and multidisciplinary support. Furthermore, the association we observed between HM feeding and reduced GF further underscores the importance of robust lactation support and policies promoting the sustained availability of human milk for this vulnerable population.

While the application of the latest ESPGHAN definitions and Intergrowth 21st standards ensure high clinical relevance and reproducibility, certain limitations must be acknowledged. The retrospective nature of this study inherently carries a risk of data gaps; however, our missing rate for the included variables remained notably low (approximately 3%). Nevertheless, complete data on certain maternal covariates (specifically pre-pregnancy BMI, gestational diabetes, and ethnicity) were not consistently available in the historical records, preventing their inclusion as potential confounders. Furthermore, the relatively small sample size of the GR subgroup constrained our ability to conduct robust multivariate regression analyses for this specific cohort.

## 5. Conclusions

In conclusion, our study demonstrates that, despite advances in neonatal care, nearly half of VLGA/VLBW infants experience significant GF before discharge. By adopting the ESPGHAN framework, we identified that ELBW infants, male infants, those with severe BPD, and those with significant postnatal weight loss are at the highest risk for GF. Crucially, the combination of GR at birth and postnatal GF creates a “perfect storm” that almost inevitably leads to undernutrition. These findings emphasize that GF at discharge is not a monolithic outcome but rather the result of distinct clinical pathways, necessitating a more refined approach to nutritional monitoring and intervention in the VLBW population.

The identification of GF and UN at discharge is only the beginning. The next crucial step is to evaluate how different growth “phenotypes” influence long-term growth trajectories and neurodevelopmental outcomes during the first years of life. Standardizing these definitions in the NICU will allow us to build more predictable models for post-discharge recovery and ensures that the most vulnerable infants receive the targeted nutritional support they need during the critical window of development.

## Figures and Tables

**Table 1:** Potential predictors of growth faltering at discharge. Values are express as median (interquartile range) or number [percentage] as appropriate.

	Growth Faltering (n = 182)	Adequate Growth (n = 220)	*p*
*Prenatal data*			
Maternal hypertension	39/173 [22.5]	60/203 [29.6]	0.129
Antenatal steroids	148/170 [87.1]	181/204 [88.7]	0.636
Antenatal MgSO_4_	59/156 [37.8]	55/187 [63.2]	0.108
IUGR	32/157 [20.4]	56/187 [30.0]	**0.048**
Twin status	56/182 [30.8]	80/220 [36.4]	0.246
*Perinatal data*			
Vaginal delivery	71/181 [39.2]	75/220 [34.1]	0.299
Gestational age (weeks)	29.00 (4.93)	31.00 (2.68)	**<0.001**
Birth weight (g)	1156 (602)	1339.5 (318)	**<0.001**
ELBW	72/182 [39.6]	39/220 [17.7]	**<0.001**
Growth restriction at birth	23/182 [12.6]	52/220 [23.6]	**0.005**
SGA < 3rd centile	9/182 [4.9]	26/220 [11.8]	**0.020**
Birth length (cm)	37 (7)	39 (4)	**<0.001**
Birth head circumference (cm)	27 (5)	28 (2.5)	**<0.001**
Sex (female)	71/182 [39.0]	122/220 [55.5]	**0.001**
*Neonatal data*			
Weight loss after birth (max value-%)	12.40 (8.02)	10.40 (6.61)	**<0.001**
IVH	58/182 [31.9]	49/220 [22.3]	**0.032**
PVL	10/180 [5.6]	4/219 [1.8]	0.056
EOS	10/181 [5.5]	11/220 [5.0]	0.826
LOS	42/181 [23.2]	20/220 [9.1]	**<0.001**
BPD	50/182 [27.5]	20/219 [9.1]	**<0.001**
PDA requiring treatment	59/182 [32.4]	40/220 [18.2]	**0.001**
NEC stage ≥ 2	10/182 [5.5]	3/219 [1.4]	**0.024**
Human milk at discharge	129/181 [71.3]	187/219 [85.4]	**<0.001**
Length of hospital stay (days)	57 (50)	34 (22)	**<0.001**
Post-menstrual age at discharge (weeks)	37.14 (4.00)	35.85 (2.15)	**<0.001**

IUGR: intrauterine growth restriction; ELBW: extremely low birth weight; SGA: small for gestational age; IVH: intraventricular hemorrhage; PVL: periventricular leukomalacia; EOS: early onset sepsis; LOS: late onset sepsis; BPD: bronchopulmonary dysplasia, PDA: patent ductus arteriosus; NEC: necrotizing enterocolitis.

**Table 2:** Regression model evaluating the relationship between neonatal variables and growth faltering at discharge.

	B	S.E.	Exp (B)	*p*
ELBW	0.500	0.316	1.648	0.114
GR at birth	−0.572	0.337	0.564	0.890
Sex	−1.000	0.243	0.368	**<0.001**
IVH	−0.026	0.276	0.974	0.924
LOS	0.690	0.359	1.994	0.055
BPD	0.742	0.371	2.101	**0.045**
PDA	0.204	0.298	1.227	0.493
NEC stage ≥ 2	0.730	0.864	2.076	0.398
Weight loss	0.060	0.021	1.062	**0.005**
HM at discharge	−0.906	0.300	0.404	**0.003**
Constant	−0.065	0.409	0.937	0.874

ELBW: extremely low birth weight; GR: growth restriction; IVH: intraventricular hemorrhage; LOS: late onset sepsis; BPD: bronchopulmonary dysplasia; PDA: patent ductus arteriosus; NEC: necrotizing enterocolitis; HM: human milk.

**Table 3:** Regression model evaluating the relationship between neonatal variables and undernutrition at discharge in infants with an adequate nutritional status at birth.

	B	S.E.	Exp (B)	*p*
Maternal hypertension	1.254	0.401	3.505	**0.002**
ELBW	0.811	0.388	2.250	**0.037**
Sex	−0.550	0.344	0.577	0.110
LOS	0.250	0.398	1.284	0.530
BPD	0.425	0.425	1.529	0.317
HM at discharge	−0.233	0.370	0.800	0.546
GF at discharge	2.368	0.452	10.678	**<0.001**
Constant	−3.344	0.593	0.035	<0.001

ELBW: extremely low birth weight; LOS: late onset sepsis; BPD: bronchopulmonary dysplasia; HM: human milk; GF: growth faltering.

## Data Availability

The data presented in this study are available on request from the corresponding author due to ethical restrictions.

## Abbreviations

The following abbreviations are used in the manuscript:

BPD	bronchopulmonary dysplasia
BW	birth weight
EOS	early onset sepsis
ESPGHAN	European Society for Paediatric Gastroenterology, Hepatology and Nutrition
GA	gestational age
GF	growth faltering
GR	growth restriction
HC	head circumference
HM	human milk
IQR	interquartile range
IUGR	intrauterine growth restriction
IVH	intraventricular hemorrhage
LFA	length for age
LOS	late onset sepsis
NEC	necrotizing enterocolitis
NICU	neonatal intensive care unit
PDA	patent ductus arteriosus
PMA	postmenstrual age
PVL	periventricular leukomalacia
SD	standard deviation
SGA	small for gestational age
UN	undernutrition
VLBW	very low birth weight
VLGA	very low gestational age
WFA	weight for age
